# Prognostic value of cough force measured by peak expiratory flow in a 4-year longitudinal cohort study of geriatric patients with oropharyngeal dysphagia

**DOI:** 10.3389/fragi.2025.1512813

**Published:** 2025-06-17

**Authors:** Kiril Stoev, Rainer Wirth, Bendix Labeit, Paul Muhle, Sonja Suntrup-Krueger, Rainer Dziewas, Gero Lueg, Ulrike Sonja Trampisch, Maryam Pourhassan

**Affiliations:** ^1^ Department of Geriatric Medicine, Marien Hospital Herne, Ruhr University Bochum, Herne, Germany; ^2^ Department of Neurology, University Hospital Düsseldorf, Düsseldorf, Germany; ^3^ Department of Neurology, University Hospital Münster, Münster, Germany; ^4^ Department of Neurology and Neurorehabilitation, Klinikum Osnabrück, Osnabrück, Germany

**Keywords:** oropharyngeal dysphagia, cough force, pneumonia, peak expiratory flow, mortality, older adults

## Abstract

**Introduction and objective:**

Oropharyngeal dysphagia (OD) is a potentially life-threatening disorder of the swallowing process that may significantly impair a patient’s prognosis and quality of life. This study aimed to investigate the association between cough force (measured by peak expiratory flow) and pneumonia incidence in older hospitalized patients with OD and to assess the relationship between peak flow, dysphagia severity and mortality over a 4-year follow-up period.

**Methods:**

In this retrospectively longitudinal cohort study, OD was evaluated using flexible endoscopic examination of swallowing (FEES). Patients with suspected OD underwent Peak Flow (PF) measurement prior to initiation of FEES. Follow-up data were collected on pneumonia incidence, episodes, and patient survival via telephone surveys. Cox regression models, adjusted for potential confounding variables such as age and gender, were used to explore the relationship between pneumonia incidence, PF and dysphagia severity.

**Results:**

Among 98 patients (mean age 80.4 ± 8.2 years, 67% male), the median PEF was 220 L/min (IQR 150–300). Post-discharge, 38% developed pneumonia—11% had one episode and 27% had multiple episodes. Dysphagia severity was mild in 40%, moderate in 40%, and severe in 20% of patients. Over an average follow-up of 1,334 days (3.7 years), the mortality rate was 64%. Patients with lower PF experienced a significantly higher risk of developing pneumonia compared to those with higher PF (p = 0.030). Patients with severe dysphagia had a substantially lower survival rate compared to those with light or moderate dysphagia, as demonstrated by the Cox-models.

**Conclusion:**

Reduced cough force as measured by peak expiratory flow was significantly associated with an increased risk of pneumonia in older hospitalized patients with OD.

## Introduction

Oropharyngeal dysphagia (OD) is a potentially life-threatening disorder of the swallowing process that may significantly impair a patient’s prognosis and quality of life (Cosentino et al., 2022; Panebianco et al., 2020). Dysphagia can affect all components of swallowing and is frequently associated with severe complications such as aspiration pneumonia, malnutrition, dehydration, hospitalisations and increased mortality (Panebianco et al., 2020; Labeit et al., 2023a).

Various neurological diseases dominate the etiology of OD (Panebianco et al., 2020; Khan et al., 2014), but anatomical changes of the larynx and pharynx, including cancer are so relevant (Chheda, 2022; Kuhn et al., 2023). The pathophysiology of OD is complex and not yet fully understood (Dziewas et al., 2017). Representing its complexity, swallowing involves multiple structures, such as various muscle groups (orofacial system, pharynx, larynx and oesophagus) (Panebianco et al., 2020), five pairs of cranial nerves (trigeminal, facial, glossopharyngeal, vagal and hypoglossal nerves) and four cervical nerves (C1-C4), all coordinated by a widespread network within the central nervous system (Labeit et al., 2023a; Christmas and Rogus-Pulia, 2019). Any disorders and anatomical changes that affect these structures may alter swallowing function (Labeit et al., 2023a; Christmas and Rogus-Pulia, 2019). The diagnosis of OD is established with videofluoroscopy (VFS) and flexible endoscopic examination of swallowing (FEES), both of which are considered to be complementary and serve as gold standards (Dziewas et al., 2016; Braun et al., 2021). However, the FEES examination does not use radiation, which allows for a more extensive and repetitive assessment of swallowing at the bedside (Dziewas et al., 2021).

For the planning of therapy and the clinical management of OD, several factors should be considered (Dziewas et al., 2024). First, the prognosis of the underlying disease plays a crucial role; for instance, dysphagia induced by a stroke may completely resolve within days or weeks, whereas dysphagia resulting from a neurodegenerative disease may progressively worsen over time (Wirth and Dziewas, 2022; Shoesmith, 2023). Second, the pattern and severity of dysphagia are of great relevance; it can vary from mild residue post-swallow to severe cases such as high-volume aspiration with every swallow (Labeit et al., 2023b; Labeit et al., 2023c). Third, the pharyngeal sensory function and the presence of a protective cough reflex, along with sufficient cough force, may be critical (Smith Hammond et al., 2009; Choi et al., 2021). Impaired cough reflex and/or insufficient cough force can prevent adequate airway clearance after aspiration, leading to bronchial inflammation, aspiration pneumonia and respiratory failure (Choi et al., 2021). Some efforts have been made to standardize cough reflex testing (Field et al., 2018; Aviv, 2000), which however remains challenging in clinical routine. Instead of cough reflex testing, most clinicians assess pharyngeal and laryngeal sensitivity during endoscopic examination as a surrogate for the sensory part of the protective cough reflex (Aviv, 2000; Kaneoka et al., 2015). However, one important prognostic factor that has not yet been sufficiently studied is the cough force as the motor part of the protective cough reflex, which may play a major role in airway clearance, particularly in older patients with sarcopenia and frailty.

Given the fact that OD is associated with reduced cough force (Han et al., 2020), the question arises if the risk of aspiration pneumonia increases (Panebianco et al., 2020; Christmas and Rogus-Pulia, 2019) if cough force is low, because patients are unable to clear secretions and foreign material from the throat and airways when coughing (Labeit et al., 2023a; Pitts et al., 2010). This impairment may be relevant for both, voluntary coughing and reflexive cough, and is explained by weak respiratory muscles and impaired glottis function (Cecconi and Di Piero, 2012). Conversely, enhancing the function of the respiratory muscles and glottis may positively affect OD (Cecconi and Di Piero, 2012).

Although there has been limited evidence regarding the relevance of cough force and methods to measure it (Tzani et al., 2014; Kulnik et al., 2016), it seems plausible that this physiological function is significant for the prognosis of geriatric patients with dysphagia and measuring cough force could therefore potentially help in decision-making regarding safe feeding strategies. Therefore, the primary objective of this study is to investigate the association between cough force, as measured by peak expiratory flow, with the general prognosis and the incidence of pneumonia in a clinically and endoscopically well-characterized cohort of older hospitalized patients with OD. The secondary objective is to evaluate the relationship between peak flow, dysphagia severity and mortality over the follow-up period.

## Subjects and methods

This retrospective longitudinal cohort study was conducted at the geriatric acute care unit of Marien Hospital Herne, University Hospital of Ruhr-University Bochum, Germany, with baseline data collected retrospectively. All patients undergoing FEES-examinations and consecutively admitted to the geriatric ward between December 2018 and January 2020 were included. In December 2018, we introduced a routine measurement of peak expiratory flow directly before every FEES. However, due to the coronavirus pandemic, examinations of expiratory peak flow (PF) were discontinued in January 2020 for hygiene reasons. Inclusion criteria comprised individuals aged 65 years and older with documented FEES, along with documented PF measurement prior to the FEES examination, and a diagnosis of OD during FEES. Exclusion criteria were incomplete data including follow-up and decline of participation in the written follow-up questionnaire or follow-up phone call. The results of FEES examinations, PF measurements, diagnoses, and geriatric assessment were retrospectively retrieved from the hospital information system at baseline. The primary outcome was the incidence of pneumonia during the follow-up period, while the secondary outcome was mortality.

### Geriatric assessment

During the initial phase of hospitalization, all study participants underwent a comprehensive geriatric assessment. Nutritional status was evaluated using the Mini Nutritional Assessment short-form (MNA-SF) (Kaiser et al., 2009), which categorizes patients into three groups: normal nutritional status (12–14 points), at-risk of malnutrition (8–11 points), and malnourished (0–7 points). Functional capability in activities of daily living was assessed using the German version of the Barthel Index (BI) (Mahoney and Barthel, 1965), which operates on a scale of 0–100, with higher scores indicating greater independence. Frailty was evaluated using the FRAIL scale (Morley et al., 2012) with scores of 1-2 indicating pre-frailty and 3–5 indicating frailty. The risk of sarcopenia was determined through the SARC-F questionnaire (Drey et al., 2020), with scores ≥4 suggesting probable sarcopenia. The Charlson Comorbidity Index (CCI) (Charlson et al., 1987) was utilized to classify the number and severity of medical comorbidities. Handgrip strength was measured three times on the dominant or unaffected side, with the maximum score recorded. Cognitive status was assessed using the Montreal Cognitive Assessment (MoCA) (Nasreddine et al., 2005). In persons where the MoCA assessment was incomplete due to non-cognitive reasons, such as severe visual impairment, the result was extrapolated based on the number of questions answered. A MoCA score below 25 was categorized as cognitive impairment.

### Assessment of dysphagia

In patients with positive dysphagia screening and those with clinical signs of OD, further instrumental assessment was conducted. The FEES examination was conducted by a speech-and-language therapist under the supervision of a FEES-certified expert physician using an ENF-VH2 laryngoscope from Olympus (Hamburg, Germany) and a video documentation system from Rheder/Partner (Hamburg, Germany) following a standardized protocol.

After anatomical and functional examination and evaluation of saliva management, patients were instructed to swallow fluids and food of varying consistencies. These included green-colored jelly, thickened and unthickened green-colored water, and small pieces of white bread. Each food consistency was tested three times and any occurrences of premature bolus spillage, pharyngeal residue, penetration, and aspiration, was carefully documented for each consistency. The severity of OD was graded according to Rosenbeck’s penetration-aspiration scale (PAS) (Hey et al., 2014). However, since the PAS does not evaluate premature bolus spillage and residue, severity was additionally categorized according to a more recently introduced FEES dysphagia severity score with four different levels of severity (Warnecke et al., 2016; Warnecke et al., 2010). Grade 0 denoted no clinically relevant neurogenic dysphagia, grade 1 represented the mildest form of neurogenic dysphagia (premature bolus spillage and/or significant residue, but no penetration or aspiration), grade 2 indicated moderate neurogenic dysphagia with penetration or aspiration of one food consistency, and grade 3 indicated severe neurogenic dysphagia with penetration or aspiration of two or more food consistencies. In a prospective multicentre study this scoring system matched well with the Functional Oral Intake Scale derived from FEES (Dziewas et al., 2019).

All patients diagnosed with OD received individualized dysphagia therapy provided by a speech-and-language therapist during their hospital stay. The therapeutic approach was tailored to each patient based on the findings of the FEES examination, in accordance with established clinical practice guidelines.

### Assessment of cough force

The force of cough thrust is closely associated with PF during forced expiratory manoeuvres (Suárez et al., 2002). Because it is easier to execute, in particular for geriatric patients, we therefore measured peak expiratory flow as a surrogate. For this study, we employed the Vitalograph PF meter (Model 4,300, Vitalograph, Ennis, Ireland) to measure peak expiratory flow. Prior to undergoing FEES, each patient was instructed to achieve maximal lung inflation by taking a deep, full breath. After securely placing and sealing the mouthpiece with their lips and nose instrumentally pinched, the patient was then directed to exhale as forcefully as possible in a single, maximum effort. To enhance the reliability of the measurement, three attempts per patient were performed. The highest of these three values was then recorded as PF value. According to the manufacturer, the device is designed to measure flows ranging from 50 to 800 L/min with a specified accuracy of ±10 L/min or ±10% of the measured value.

### Follow-up

Follow-up data were gathered via a telephone survey conducted with the patient, their relatives, or their general practitioner. Baseline data were collected retrospectively as part of standard clinical care, and written informed consent was waived for this retrospective data collection and for participation in the telephone follow-up. Prior consent for participation in the telephone follow-up was secured through a consent form, which was mailed to the patient’s home. Patients were informed about the upcoming telephone contact as part of the consent process. In instances where no response was received to the mailed consent form, verbal consent was obtained over the phone before proceeding with the survey. During the telephone follow-up survey, patients or their caregivers were asked about the occurrence of pneumonia following discharge from the hospital stay. If applicable, the patient’s date of death was also recorded. When patients or their relatives could not be reached or provided unreliable information, the patient’s family doctor was consulted. If uncertainties persisted regarding survival status, verification was sought from the residents’ registration office as needed. In addition, the patient’s records in the hospital information system were evaluated regarding any pneumonia that had been treated and death in our hospital since the baseline assessment.

### Statistical analysis

Statistical analyses were conducted using SPSS software (SPSS Statistics for Windows, Version 29.0, IBM Corp., Armonk, NY, United States). Continuous variables were expressed as means and standard deviations (SDs) for normally distributed data, and median values with interquartile ranges (IQR) for non-normally distributed data. Categorical variables were presented as absolute numbers and relative frequencies (%). To investigate the association between mortality and pneumonia incidence with PF, Cox proportional hazard models were employed. These models were adjusted for potential confounders such as age, gender, dysphagia severity, and hand grip strength, providing 95% confidence intervals (CI) for hazard ratios (HRs). Cox proportional hazards model survival curves and pneumonia-free probability curve from cox proportional hazards model were generated to illustrate the cumulative incidence of survival and pneumonia-free probability following FEES, stratified by key variables such as PF levels and dysphagia severity. This stratification allowed for a detailed analysis of how specific clinical parameters influenced patient outcomes over time. The significance of the results was determined using Wald tests in the multivariate Cox models. In addition, to assess potential multicollinearity between independent variables included in the Cox models, Variance Inflation Factors (VIFs) were calculated, with VIF values less than 2 generally indicating a low level of multicollinearity. A P value <0.05 was accepted as the limit of significance.

## Results

Baseline characteristics of study participants are presented in Table 1. A total of 99 patients were initially included in the database. Follow-up data were successfully obtained for 98 of these patients. One patient was lost to follow-up due to a complete lack of contact information, including from the responsible general practitioner.

**TABLE 1:** Characteristics of study population upon admission.

	All (n = 98)
Gender (n, %)	
Females	32 (33)
Males	66 (67)
Age (yrs), mean ± SD	80.4 ± 8.2
Height (cm), mean ± SD	168.8 ± 8.9
Body weight (kg), mean ± SD	80.7 ± 7.7
BMI (kg/m^2^), mean ± SD	25.7 ± 4.4
Geriatric assessments, Median (IQR)	
MNA-SF	8 (6–10)
Normal nutritional status (n, %)	7 (8)
At risk of malnutrition (n, %)	44 (49)
Malnourished (n, %)	39 (43)
Barthel-Index	45 (35-60)
Frail Simple score	4 (3-4)
SARC‐F scores	7 (5–8)
Cognitive function (MoCA)	19 (16–23)
Charlson Comorbidity Index	9 (7–12)
Handgrip strength (kg)	18.5 (1–28)
Peak flow (l/min), Median (IQR)	220 (150–300)
PAS, Median (IQR)	2 (1–3)
PAS 1-3	79 (81)
PAS 4-8	19 (19)
Dysphagia severity (n, %)	
Mild	39 (40)
Medium	39 (40)
Severe	20 (20)
Pneumonia (n, %)	
Yes	37 (38)
No	61 (62)
Number of pneumonia (n, %)	
0	61 (62)
1	11 (11)
≥2	26 (27)
Mortality (n, %)	63 (64)
Average time from peak flow measurement	
Days to death, mean ± SD	566.0 ± 393.6
Days to first pneumonia, mean ± SD	467.7 ± 386.0
Days to follow-up, mean ± SD	1334.2 ± 107.0

MNA-SF, mini nutritional assessment short form; MOCA, montreal cognitive assessment; PAS; Penetration aspiration scale. Values are given as number (%), mean ± SD or median (IQR, interquartile range).

The study population consisted of 98 patients with a mean age of 80.4 ± 8.2 years (67% males). Major reasons for hospital admission included falls and fractures, immobility after surgery, post-stroke care, urinary tract and pulmonary infections, heart failure and neurodegenerative diseases. According to MNA-SF, the prevalence of patients at risk of malnutrition and malnourished subjects was 49% and 43%, respectively. In addition, 82% (n = 71) of the patients were frail, 91% (n = 88) had impaired cognitive function, and 86% (n = 75) had probable sarcopenia according to SARC-F. The median PF among patients was 220 L/min (IQR 150–300). Thirty-eight percent of patients developed pneumonia post-discharge, with 11% experiencing one episode and 27% experiencing two or more episodes. Regarding dysphagia severity, 40% of patients were classified with mild and medium severity each, while 20% exhibited severe dysphagia. The mortality rate during a mean follow-up of 1,334 days (3, 7 years) was 64%. From the time of PF measurement, the average time to death was 566 days, and to the first pneumonia 468 days. Furthermore, the primary etiologies of OD in our cohort were neurological and neurodegenerative conditions, including Parkinson’s disease, ischemic and haemorrhagic stroke, dementia, delirium and rare causes such as amyotrophic lateral sclerosis (ALS) and prior radiotherapy (n = 3). Table 2 presents the results from three Cox regression models examining the association of mortality with PF, adjusted for potential confounders such as age, gender, dysphagia severity, and hand grip strength. In Model 1, there is a significant association between mortality and PF (p = 0.042), indicating that lower PF values are associated with higher mortality risk. Gender also shows a significant effect (p = 0.031), with females having a higher risk of mortality compared to males. Age does not show a significant association with mortality in this model (p = 0.118). Model 2 includes dysphagia severity along with the previously considered variables. Dysphagia severity shows a significant association with increased mortality risk (p = 0.030). Both age (p = 0.047) and female gender (p = 0.018) are significant predictors of increased mortality risk. Model 3 further includes hand grip strength; in this model, neither PF (p = 0.106) nor hand grip strength (p = 0.373) shows significant associations with mortality. Dysphagia severity and female gender show trends but do not reach significance levels. In addition, we used PAS score instead of dysphagia severity in the analyses and we found nearly the same results with different values.

**TABLE 2:** Association of mortality with peak flow and dysphagia according to Cox regression models adjusted for potential confounders in total population.

	Model 1		
	*P*	HR	95% CI
Peak flow	0.042	0.997	0.994–1.000
Age	0.118	1.025	0.994–1.058
Gender (female)	0.031	0.522	0.289–0.942

	Model 2		
	*P*	HR	95% CI
Peak flow	0.081	0.997	0.994–1.000
Age	0.047	1.032	1.000–1.064
Gender (female)	0.018	0.482	0.264–0.880
Dysphagia severity	0.030	1.460	1.038–2.052

	Model 3		
	*P*	HR	95% CI
Peak flow	0.106	0.997	0.994–1.001
Age	0.299	1.021	0.982–1.062
Gender	0.061	0.537	0.280–1.029
Dysphagia severity	0.061	1.421	0.983–2.053
Hand grip strength	0.373	0.985	0.952–1.019

HR, hazard ratio; CI, confidence interval. Peak flow and hand grip strength were added into the model as continuous variable, dysphagia was categorized as mild, moderate or severe.

The results from Cox regression models examining the association of pneumonia incidence with PF and dysphagia severity, adjusted for potential confounders such as age, gender, dysphagia severity, and hand grip strength are presented in Table 3. Model 1 shows that lower PF is significantly associated with an increased risk of pneumonia (p = 0.030). Gender also significantly affects pneumonia risk, with females having a higher risk compared to males (p = 0.002). Additionally, older age is significantly associated with an increased risk of pneumonia (p = 0.049). Model 2 additionally includes dysphagia severity, where lower PF continues to be significantly associated with higher pneumonia risk (p = 0.024), and the protective effect of being male remains strong (p = 0.001). Dysphagia severity does not significantly influence pneumonia risk (p = 0.342), while age continues to be a significant risk factor (p = 0.030). Model 3 adds hand grip strength to the analysis, where PF continues to be a significant predictor (p = 0.012), and dysphagia severity and hand grip strength still do not present significant associations. Both age (p = 0.028) and female gender (p = 0.013) continue to signify higher risks of pneumonia.

**TABLE 3:** Association of pneumonia (yes/no) with peak flow and dysphagia according to Cox regression models adjusted for potential confounders in total population.

	Model 1		
	*P*	HR	95% CI
Peak flow	0.030	0.995	0.991–1.000
Age	0.049	1.044	1.000–1.089
Gender (female)	0.002	0.242	0.098–0.595

	Model 2		
	*P*	HR	95% CI
Peak flow	0.024	0.995	0.991–0.999
Age	0.030	1.049	1.005–1.094
Gender (female)	0.001	0.222	0.088–0.561
Dysphagia severity	0.342	1.251	0.789–1.984

	Model 3		
	*P*	HR	95% CI
Peak flow	0.012	0.994	0.989–0.999
Age	0.028	1.059	1.006–1.115
Gender (female)	0.013	0.250	0.084–0.746
Dysphagia severity	0.373	1.263	0.756–2.111
Hand grip strength	0.849	1.004	0.965–1.044

HR, hazard ratio; CI, confidence interval. Peak flow and hand grip strength were added into the model as continuous variable, dysphagia was categorized as mild, moderate or severe.

**FIGURE 1:**
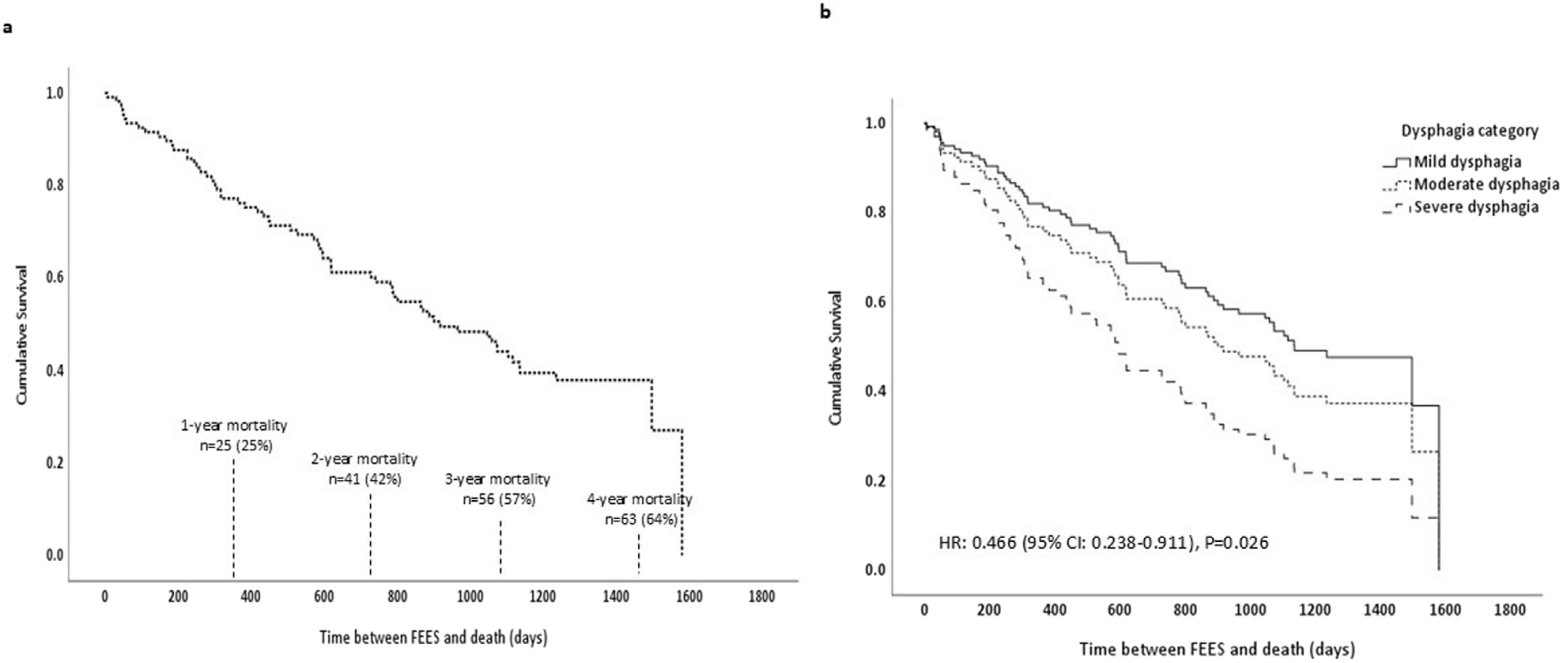
Cox proportional hazards model survival curves. **(a)** Shows the survival curve for the entire cohort including mortality rates. **(b)** Shows survival curves by dysphagia severity: mild dysphagia (solid line), moderate dysphagia (dotted line), and severe dysphagia (dashed line), along with the associated hazard ratio and P value; FEES, Flexible Endoscopic Evaluation of Swallowing.

Figure 1 illustrates Cox proportional hazards model survival curves across two panels, detailing the mortality outcomes for the entire cohort following FEES. Figure 1A demonstrates the overall survival curve for the cohort, showing a significant decline in survival rates over time. Specifically, within the first year post-FEES, 25% of the cohort had deceased (n = 25), escalating to 42% by the second year (n = 41), 57% by the third year (n = 56), and reaching 64% mortality by the fourth year (n = 63). Figure 1B stratifies the cohort by dysphagia severity, demonstrating the profound impact of dysphagia severity on survival. It clearly shows that patients with severe dysphagia have a substantially lower survival rate compared to those with mild or moderate dysphagia, as demonstrated by the Cox-models.

**FIGURE 2:**
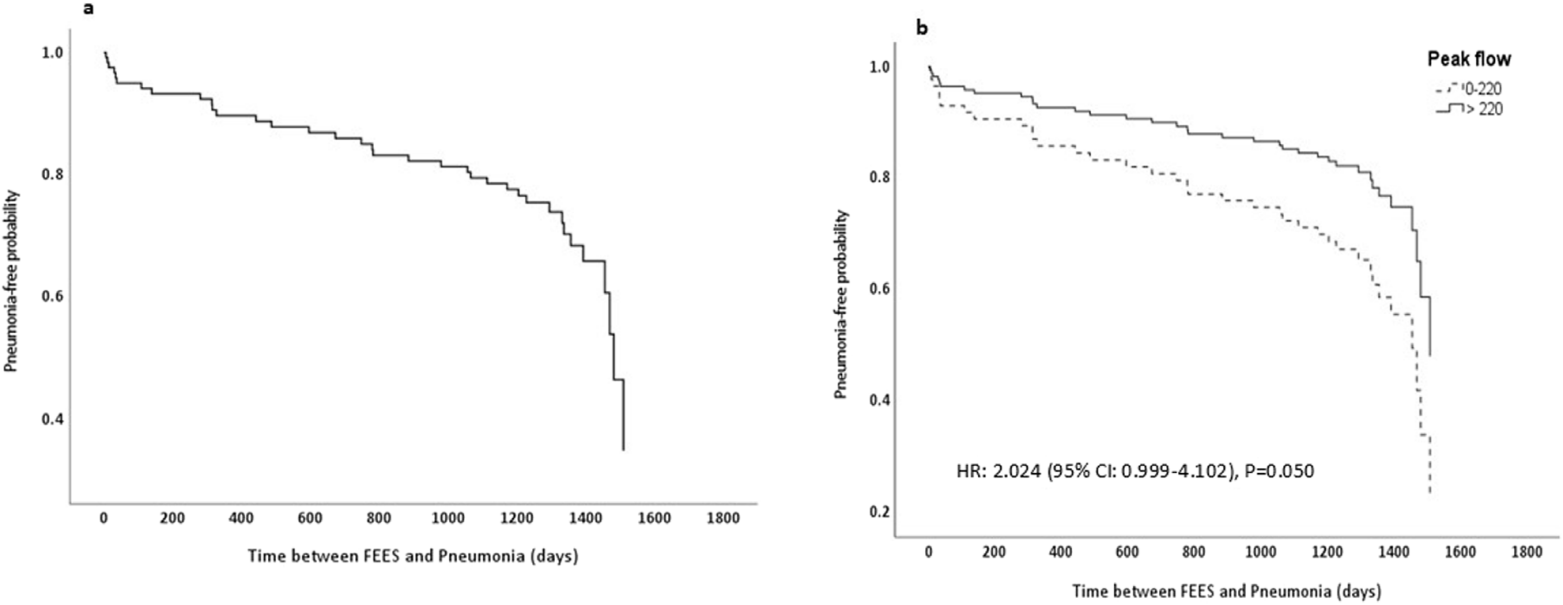
Pneumonia-free probability curve from cox proportional hazards model. **(a)** Displays pneumonia-free probability for the entire cohort. **(b)** Stratifies the cohort by peak flow rates, with group 1 (0–220 L/min) shown as a dashed line and group 2 (>220 L/min) as a solid line, along with the associated hazard ratio and P value; FEES, Flexible Endoscopic Evaluation of Swallowing.

Figure 2 displays the pneumonia-free probability curves derived from a Cox proportional hazards model after FEES. Figure 2A demonstrates the overall pneumonia-free probability across the entire cohort, showing a progressive decline over time which indicates the consecutive occurrence of pneumonia. Figure 2B stratifies the cohort based on PF rates, comparing those with lower rates (≤220 L/min, shown as a dashed line) against those with higher rates (>220 L/min, shown as a solid line). The survival curves in this panel reveal that patients with lower PF experience a significantly higher risk of developing pneumonia compared to those with higher PF.

## Discussion

The role of cough force as a protective mechanism against aspiration and aspiration pneumonia in patients with OD has not been well-documented in older patients. However, due to the high prevalence of sarcopenia and weakness as well as sensory loss in the older population, this is of particular interest. Our findings highlight that reduced cough force, as measured by PF, is significantly associated with an increased risk of pneumonia, even if the calculation is adjusted for age, gender and dysphagia severity. To investigate, if this association is independent from general muscle weakness, we also included hand grip strength in the model and still expiratory peak flow was significantly associated with the occurrence of pneumonia during follow-up in geriatric patients with OD. However, not PF, but dysphagia severity emerged as a predictor of increased mortality risk in this population.

Cough force has been shown to correlate with the risk of pneumonia in a small study of 55 unselected patients with dysphagia (Bianchi et al., 2012). Kulnik et al. (2016) demonstrated in stroke patients that each 1 L/min increase in voluntary peak cough flow corresponded to a 0.6% reduction in pneumonia risk, based on logistic regression analysis. In a study by Smith Hammond et al. (2009) voluntary peak cough flow was used to predict aspiration risk in ischemic stroke patients, revealing that a peak cough flow below 174 L/min significantly increased the likelihood of aspiration. Similarly, Sakai et al. (2020) indicated that peak cough flow is associated with aspiration risk during an endoscopic swallowing assessment in older adults with community-acquired pneumonia, identifying a cut-off value of 190 L/min. In our study, we intentionally chose to use peak expiratory flow instead of peak cough flow for several reasons. Peak expiratory flow is easier to measure, especially in geriatric patients who may have limited ability to cooperate during testing. Additionally, peak expiratory flow is widely used in routine clinical practice, making it a more practical choice for our purpose. Although peak cough flow typically yields higher values than peak expiratory flow, the two measures are highly correlated, ensuring that peak expiratory flow remains a reliable and valid indicator in this context (Suárez et al., 2002; Fitzgerald et al., 2024).

The results of our study revealed that lower PF is significantly associated with an increased risk of pneumonia (p = 0.030). Stratification of our cohort based on PF rates showed that patients with lower PF rates than the median value of 220 L/min had a significantly higher risk of developing pneumonia compared to those with higher PF rates (>220 L/min). Intuitively, effective coughing requires a certain force to expel aspirated material, which is crucial for maintaining airway safety. However, in our study, we aimed to validate this association in older patients with dysphagia and to identify a critical threshold value for this force. Considering the delicate balance between swallowing safety and the ability to maintain oral feeding, PF could play a pivotal role in stratifying pneumonia risk and guiding optimal feeding strategies. If our findings are corroborated by further studies, PF could become an essential tool in clinical decision-making for this vulnerable population. In addition, our data show that expiratory peak flow and expiratory muscle strength are an important focus of dysphagia treatment, as addressed by expiratory muscle strength training (EMST) (Brooks et al., 2019).

Dysphagia is well-documented as a major risk factor for pneumonia in older individuals across various studies. One study reported that around one-third of older adults with dysphagia developed aspiration pneumonia during rehabilitation, while another study found a prevalence of 48.2% among patients with dysphagia (Langmore et al., 2002; Bosch et al., 2023). Although the exact figures differ across studies, it is well-established that individuals with dysphagia are at significantly increased risk for aspiration pneumonia compared to those without swallowing impairments. However, our findings differ from these previous studies regarding the relationship between dysphagia and pneumonia. Our data appear to indicate that dysphagia severity may not significantly influence pneumonia risk. We think that this discrepancy emerges due to several factors (Ebihara, 2022), in particular features and the size of the cohort.

Another important finding of the current study is the identification of dysphagia severity as a critical predictor of mortality. Patients with severe dysphagia exhibited substantially lower survival rates compared to those with mild or moderate dysphagia. Initially, we observed a significant association between mortality and PF values (p = 0.042), with lower PF values linked to higher mortality risk. However, when dysphagia severity was included in the model, the effect of PF disappeared. This suggests that dysphagia severity may be a more direct and influential factor in predicting mortality, overshadowing the impact of PF. Supporting this, a study on patients with head and neck cancer found that the most severe dysphagia, which was associated with lower survival rates, was the strongest independent predictor of survival in these patients (Shune et al., 2012).

In the current study, the overall survival analysis indicated a significant decline over time, with 25% of the cohort deceased within the first year post-FEES, escalating to 64% by the fourth year. This trend underscores the severity and frequently progressive nature of diseases associated with dysphagia and the importance of early intervention and continuous management to improve patient outcomes. Several studies have demonstrated a strong association between dysphagia and increased mortality in older adults. A prospective cohort study involving 391 older adults in intermediate care units reported a 25% mortality rate within 1 year, with swallowing dysfunction independently associated with mortality (adjusted HR: 1.67, 95% CI 1.02–2.75; P = 0.041) (Hägglund et al., 2019). Additionally, Carrion and colleagues identified (Carrión et al., 2015) oropharyngeal dysphagia as a significant risk factor for 1-year mortality in hospitalized older adults, independent of factors such as functional status, malnutrition, and comorbidities. Their study demonstrated that patients with oropharyngeal dysphagia had a 1.7-fold higher risk of 1-year mortality compared to those with normal swallowing function (Carrión et al., 2015). These findings emphasize the need for comprehensive assessment and targeted interventions for patients with dysphagia to mitigate the associated mortality risk.

The novelty of this study lies in the evaluation of cough force, as measured by peak expiratory flow, as a prognostic tool for pneumonia and mortality in older hospitalized patients with OD. While previous studies have focused on cough force in other populations or used different methods, our study uniquely assesses this association in a well-characterized cohort of older adults using a practical, clinically applicable method (PF). Furthermore, the longitudinal design with a long follow-up period provides valuable insights into the progression of OD-related complications, setting this study apart from shorter-term investigations. However, this study has several limitations. The retrospective nature of the study introduces inherent biases, though it offers a foundational basis for future prospective research. The sample size was restricted to 98 patients due to COVID-19 pandemic-related hygiene measures, limiting the generalizability of our findings. Additionally, the average MoCA score of 19 among participants indicates decreased cognitive function, which may have impacted their ability to fully cooperate for evaluation of peak expiratory flow. To mitigate this, pre-assessment education and practice were provided to ensure patient comprehension. Although a strong correlation between peak expiratory flow and voluntary cough strength has been demonstrated in previous studies, it is important to note that the strength of a cough in response to aspiration reflects a reflexive, rather than a voluntary task. This reflexive action involves not only motor responses, as assessed in this study, but also incorporates sensory components, which are not directly evaluated here. Furthermore, the incidence of pneumonia was self-reported by patients or their caregivers during telephone follow-ups. Although efforts were made to confirm these reports by consulting clinical records and, where possible, verifying with general practitioners, no imaging data (e.g., chest X-rays) were consistently available to validate pneumonia diagnoses. Lastly, it is important to consider that peak expiratory flow may influence pneumonia incidence independently of cough strength, e.g., higher peak flow rates may be associated with better ventilation and thus reduced accumulation of respiratory bacteria. Future studies are needed to confirm these findings in larger, prospective cohorts and in particular in patients with and without aspiration during swallowing assessment.

## Conclusion

The results of this study indicate that reduced cough force as measured by peak expiratory flow is significantly associated with an increased risk of pneumonia in older hospitalized patients with OD.

## Data Availability

Additional data are available from the corresponding author on reasonable request.
